# Intensive versus standard blood pressure control in older persons with or without diabetes: a systematic review and meta-analysis of randomised controlled trials

**DOI:** 10.1177/01410768231156997

**Published:** 2023-02-24

**Authors:** Samuel Seidu, Harini Willis, Setor K Kunutsor, Kamlesh Khunti

**Affiliations:** 1 Diabetes Research Centre, College of Medicine, Biological Sciences and Psychology, University of Leicester, Leicester, LE5 4PW, UK; 2 NIHR Applied Research Collaboration – East Midlands, Leicester, LE5 4PW, UK

**Keywords:** Diabetes, intensive blood pressure, standard blood pressure, older adults, cardiovascular disease, mortality, randomised controlled trial, systematic review, meta-analysis

## Abstract

**Objectives:**

To assess and compare the benefits and harms of intensive versus standard blood pressure (BP) control in older people with or without diabetes mellitus (DM).

**Design:**

Systematic review and meta-analysis

**Setting:**

Randomised controlled trials comparing intensive versus standard BP control, identified from MEDLINE, Embase, The Cochrane library, Web of Science and a search of bibliographies from inception till August 2022.

**Participants:**

Older people (≥65 years) with or without DM.

**Main outcome measures:**

Study-specific risk ratios (RRs) with 95% confidence intervals (CIs) were pooled for adverse vascular and safety outcomes.

**Results:**

We included six randomised controlled trials (RCTs) comprising 20,985 patients (intensive BP = 10,474 and standard BP = 10,511) with a weighted mean follow-up of 3.1 years. In the general population, the RRs (95% CIs) of intensive versus standard BP control for composite cardiovascular events or major adverse cardiovascular events (CVD/MACE), CVD mortality, coronary heart disease, stroke and heart failure were 0.71 (0.62–0.82), 0.65 (0.49–0.86), 0.75 (0.60–0.95), 0.75 (0.61–0.92) and 0.58 (0.41–0.82), respectively. Intensive BP control did not increase the risk of renal failure or serious adverse events in the general population. Two RCTs reported results for composite CVD/MACE in patients with DM with a pooled estimate of 0.85 (0.67–1.07).

**Conclusions:**

Aggregate trial evidence shows that intensive BP control (<120 to <140 mmHg) reduces the risk of adverse cardiovascular outcomes in older hypertensive patients in the general population with no increase in adverse events. Intensive BP control may confer similar benefits for older patients with DM with no evidence for harm, but this is based on limited data.

PROSPERO Registration: CRD42022349791

## Introduction

High blood pressure (BP), smoking, diabetes mellitus (DM) and abnormal lipid levels are major risk factors for cardiovascular disease (CVD), the leading cause of mortality globally. Among these modifiable risk factors, high BP or hypertension is the one with the highest prevalence and with the most comprehensive evidence for causation.^1^ The prevalence of hypertension increases linearly with age,^2^ with CVD being a major cause of mortality and morbidity in the aging population.^3^ Several observational cohort studies and landmark randomised controlled trials (RCTs) have documented the effectiveness of BP lowering for CVD and mortality prevention.^4–6^ However, the management of BP in older people is fraught with challenges because these individuals are often frail, have multiple co-morbidities and are more vulnerable to the complications of intensive BP control. The optimal BP target for cardiovascular and mortality benefit is uncertain in this population group.^7^ This is largely due to inconsistent findings from studies conducted on the topic^8–11^ and most RCTs, which inform guidelines on BP targets, rarely recruit older people.^12^ As a result, guideline recommendations differ globally. Guidelines of the American College of Physicians–American Academy of Family Physicians and the UK National Institute for Health and Care Excellence recommend a systolic blood pressure (SBP) target of <150 mmHg in older patients,^13,14^ a target of 130–139 mmHg is recommended in the 2018 European Society of Cardiology (ESC)/European Society of Hypertension (ESH) guideline,^2^ and <130 mmHg in the American College of Cardiology (ACC)–American Heart Association (AHA) guideline.^15^

Cardiovascular disease complications are the leading cause of morbidity and death in individuals with type 2 diabetes,^16^ the most common form of DM. Hypertension and DM are common conditions that co-exist, which places these individuals at substantially higher risk of adverse cardiovascular outcomes. Achieving BP control in older people with hypertension and DM is even more controversial compared with older general population participants. This is because of the limited data available on target BP levels in this population group; most landmark trials did not report age-specific results and/or excluded older patients with DM.^17^ Though a number of RCTs have demonstrated cardiovascular benefits with intensive BP control compared with standard BP control in older patients in the general population,^18^ the evidence is much limited and sparse for older patients with DM. Furthermore, the thresholds used to define intensive and standard BP control have varied across previous studies.^8,19–21^ Guideline recommendations are therefore inconsistent on by how much BP should be lowered in older individuals with DM.^17^ Given the limited and inconsistent evidence available, it would be clinically useful to quantitatively summarise the existing evidence on the impact of intensive versus standard BP control in older people and how this compares in those with and without DM. We therefore conducted a systematic review and meta-analysis of available RCTs to assess and compare the benefits and harms of intensive versus standard BP control in older people (≥65 years) with or without DM.

## Methods

### Data sources, search strategy and assessment

We prospectively registered a predefined protocol for this systematic review and meta-analysis in the PROSPERO prospective register of systematic reviews (CRD42022349791). The methodology and reporting adhered to PRISMA guidelines (Supplementary Appendix 1). We performed a systematic search, without language restriction, using MEDLINE, Embase and The Cochrane library from inception till 1 August 2022. The computer-based searches combined free texts, key words and MeSH terms related to diabetes, intensive BP, goal BP, standard BP, elderly and older patients. A MEDLINE search strategy was initially employed (Supplementary Appendix 2), which was then adapted for the other databases. One author (SKK) initially screened the titles and abstracts of the retrieved citations using Rayyan (http://rayyan.qcri.org), a free online bibliographic tool that helps to expedite the screening process using a process of semi-automation.^22^ Two authors (SS and SKK) then performed a full-text evaluation of potentially eligible articles. Each article was assessed using the inclusion criteria and any disagreement regarding the eligibility of an article was discussed, and agreement reached by consensus with a third author (HW). To identify potential studies missed by the search strategy, we performed manual searches through the reference lists of pertinent studies and reviews and citation searches using the ‘Cited Reference Search’ function in Web of Science.

### Study selection and eligibility criteria

Our eligibility criteria included the following criteria: (i) RCTs that have compared intensive BP control versus standard BP control in older adults (≥65 years), with or without DM; and (ii) reported long-term data on cardiovascular and safety outcomes. Trials that compared intensive BP control with placebo as a control arm and those that recruited specific patient populations such as those with stroke were excluded, as these trials were designed to answer separate clinical questions.

### Data extraction

A standardised predesigned data collection form, utilised in previous similar reviews,^23,24^ was used for data extraction. For each study, data regarding first author and year of publication, study design and baseline characteristics, intervention and comparator characteristics, and outcomes were extracted. One experienced reviewer (SKK) extracted the data from eligible studies and a second experienced reviewer (SS) independently double-checked the data with those in original articles.

### Outcomes

Outcomes prespecified to be evaluated included: (i) composite cardiovascular events or major adverse cardiovascular events (henceforth referred to as ‘composite CVD/MACE’); (ii) composite cardiovascular events plus renal dysfunction; (iii) other cardiovascular outcomes (CVD mortality, myocardial infarction (MI), coronary heart disease (CHD), stroke, arrhythmias and heart failure); (iv) all-cause mortality; and (v) safety outcomes of serious adverse events and renal failure. For all outcomes assessed, we accepted trial-specific definitions.

### Risk of bias and certainty of evidence

The risk of bias assessment was conducted using the Cochrane Collaboration’s risk of bias tool.^25^

This tool evaluates seven possible sources of bias: random sequence generation, allocation concealment, blinding of participants and personnel, blinding of outcome assessment, incomplete outcome data, selective reporting and other bias. For each individual domain, studies were classified into low, unclear or high risk of bias. The certainty of the body of evidence on each outcome was assessed using the Grading of Recommendations Assessment, Development and Evaluation (GRADEpro) tool (https://gdt.gradepro.org), based on study limitations, inconsistency of effect, imprecision, indirectness and publication bias.^26^ The certainty was rated at four levels: high, moderate, low and very low.

### Statistical analysis

Summary measures of effect were presented as risk ratios (RRs) with 95% confidence intervals (CIs). Measures of effect were pooled using random effects models to minimise the effect of between-study heterogeneity. Where appropriate (e.g. in the absence of substantial heterogeneity), fixed effects models were used. The standard chi-square tests and the *I*^2^ statistic were used to quantify the extent of statistical heterogeneity across studies. In our prespecified protocol, we planned to explore for sources of heterogeneity using stratified analysis and random effects meta-regression and assess for small study effects (e.g. publication bias) using formal tests such as Begg’s funnel plots^27^ and Egger’s regression symmetry test.^28^ However, we could not achieve these because each outcome was based on pooled analysis of <10 studies. To compare the effect of intensive versus standard BP control on outcomes in those with or without DM, we conducted interaction analyses using meta-regression techniques as done previously.^24^ A narrative synthesis was performed for a few studies that could not be pooled. All analyses were conducted using Stata version MP 17 (Stata Corp, College Station, TX, USA).

## Results

### Study identification and selection

**Figure 1.**
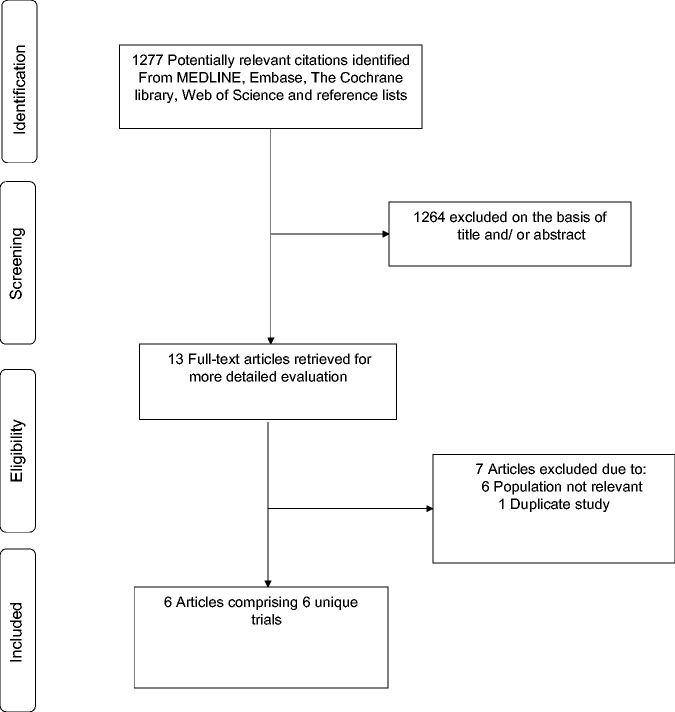
Selection of studies included in the meta-analysis.

The study selection process is presented in Figure 1. The search of relevant databases, citation checking and manual scanning of reference lists of relevant studies and reviews retrieved 1277 potentially relevant citations. Following initial screening of titles and abstracts, 1264 citations were excluded, leaving 13 articles for full text evaluation. Following detailed evaluation, seven articles were excluded. The remaining six articles based on six unique studies met the inclusion criteria and were included in the review.^9,19–21,29,30^

### Study characteristics and risk of bias

The publication dates of included articles ranged from 2008 to 2021. Relevant baseline characteristics of the individual RCTs, including BP thresholds for interventions are summarised in Table 1. The six RCTs, which were all open-labelled trials, involved 20,985 patients with diagnosed hypertension (10,474 assigned to intensive BP control and 10,511 assigned to standard BP control). All included trials used SBP thresholds; thresholds for intensive BP control group ranged from <120 to <140 mmHg and that for the standard BP control group ranged from <140 to <160 mmHg. The majority of trials were conducted in Asia (China and Japan) and two were based in the USA and Canada. All trials included patients aged ≥65 years, except for one that was based in patients aged 60–80 years with a mean age of 66.2 years.^19^ The overall weighted mean age across all trials was 71.2 years. Except for one trial that was based in patients with DM, they were all conducted in the general population; the proportion of those with DM in the general population ranged from 0 to 23%. Two trials reported subgroup results for patients with DM.^19,21^ The follow-up periods in the trials ranged from 2.0 to 4.7 years and the weighted mean follow-up was 3.1 years. All six trials reported outcome data for composite CVD/MACE (Supplementary Appendix 3). Using the Cochrane Risk of Bias tool, all six trials demonstrated a low risk of bias in random sequence generation, blinding of outcome assessment, incomplete outcome data and selective reporting, but a high risk of bias in blinding of participants and personnel (Supplementary Appendix 4).

**Table 1.** Characteristics of included trials (2008–2021).

Author, year of publication	Study name	Country	Baseline year	Population/% with DM	Age range, yrs	Male%	Threshold for intensive BP control, mmHg	Threshold for standard BP control, mmHg	Follow-up, yrs	Intensive BP controlEvents/Total	Standard BP controlEvents/Total
Goto et al., 2008^20^	JATOS	Japan	2001–2002	General population/11.8	65–85	38.9	<140	140–159	2.0	26/2212	28/2206
Ogihara, 2010^21^	VALISH	Japan	2004–2005	General population/13.0	70–85	37.5	<140	140–149	3.1	32/1545	37/1534
Cushman et al., 2010^29^	ACCORD	USA, Canada	2001/2003–2005	Diabetes/100	≥65	NR	<120	<140	4.7	20/794	23/823
Wei et al., 2013^30^	NR	China	NR	General population/23.3	>70	66.3	<140	140–149	4.0	21/363	36/361
Williamson, 2016^9^	SPRINT-Senior	USA	2010–2015	General population/0.0	≥75	62.1	<120	<140	3.1	102/1317	148/1319
Zhang et al., 2021^19^	STEP	China	2017	General population/19.1	60–80	46.5	110–129	130–149	3.3	147/4243	196/4268

ACCORD: Action to Control Cardiovascular Risk in Diabetes; BP: blood pressure; DM: diabetes mellitus; JATOS: Japanese Trial to Assess Optimal Systolic Blood Pressure in Elderly Hypertensive Patients; NR: not reported; SPRINT: Systolic Blood Pressure Intervention Trial; STEP: Strategy of Blood Pressure Intervention in the Elderly Hypertensive Patients; VALISH: Valsartan in Elderly Isolated Systolic Hypertension.

### Composite CVD/MACE

**Figure 2.**
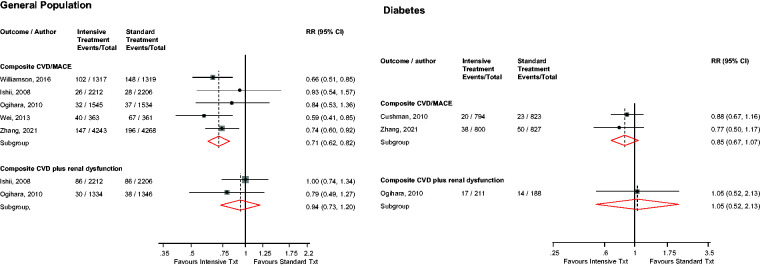
Risk for major adverse cardiovascular outcomes in older people with or without diabetes mellitus comparing intensive with standard blood pressure control.CI: confidence interval (bars); CVD: cardiovascular disease; MACE: major adverse cardiovascular events; RR: risk ratio.

In the general population, intensive versus standard BP control was significantly associated with a reduced risk of composite CVD/MACE (*n* = 5 studies): RR (95% CIs) of 0.71 (0.62–0.82; *I*^2^ = 0%; 95% CI 0–79; *p* for heterogeneity = .57) (Figure 2(a)). The results remained similar on exclusion of the trial by Zhang et al.,^19^ which recruited patients aged 60–80 years with an overall mean age of 66.2 years: RR (95% CIs) of 0.69 (0.58–0.83).

In patients with DM, intensive versus standard BP control was not significantly associated with the risk of composite CVD/MACE (*n* = 2 studies): RR (95% CIs) of 0.85 (0.67–1.07) (Figure 2(b)). There was no significant evidence of an interaction between DM status and the effect of intensive versus standard BP control on composite CVD/MACE (*p*-value for meta-regression = .21).

### Composite CVD plus renal dysfunction

In the general population, intensive versus standard BP control was not significantly associated with the risk of composite CVD plus renal dysfunction (*n* = 2 studies): RR (95% CIs) of 0.94 (0.73–1.20) (Figure 2(a)).

A single study in patients with DM showed that intensive versus standard BP control was not significantly associated with the risk of composite CVD plus renal dysfunction (Figure 2(b)).

### CVD and all-cause mortality

**Figure 3.**
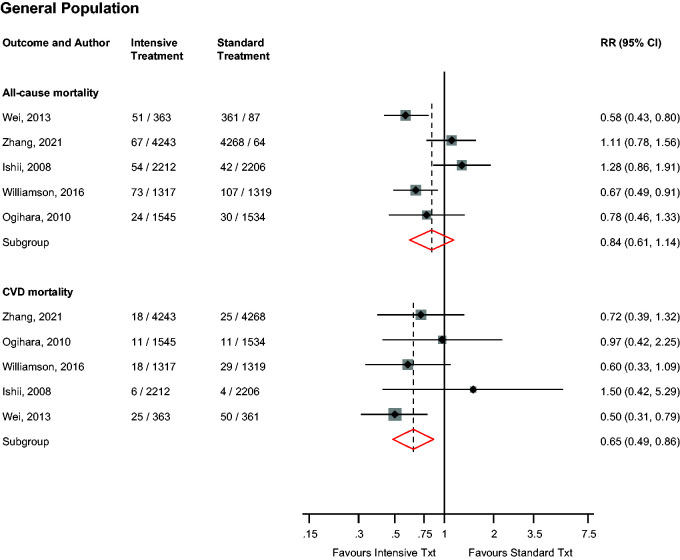
Risk for cardiovascular and all-cause mortality in older people in the general population comparing intensive with standard blood pressure control.CI: confidence interval (bars); CHD: coronary heart disease; CVD: cardiovascular disease; MACE: major adverse cardiovascular events; RR: risk ratio.

In the general population, intensive versus standard BP control was not significantly associated with the risk of all-cause mortality (*n* = 5 studies), but was significantly associated with a reduced risk of CVD mortality (*n* = 5 studies): RRs (95% CIs) of 0.84 (0.61–1.14; *I*^2^ = 72%; 95% CI 29–89; *p* for heterogeneity = .007) and 0.65 (0.49–0.86; *I*^2^ = 0%; 95% CI 0–79; *p* for heterogeneity = .42), respectively (Figure 3). On exclusion of the trial by Zhang et al.,^19^ the RRs (95% CIs) for all-cause mortality and CVD mortality were 0.78 (0.55–1.09) and 0.65 (0.44–0.96), respectively. No study evaluated these associations in patients with DM.

### Other cardiovascular outcomes

**Figure 4.**
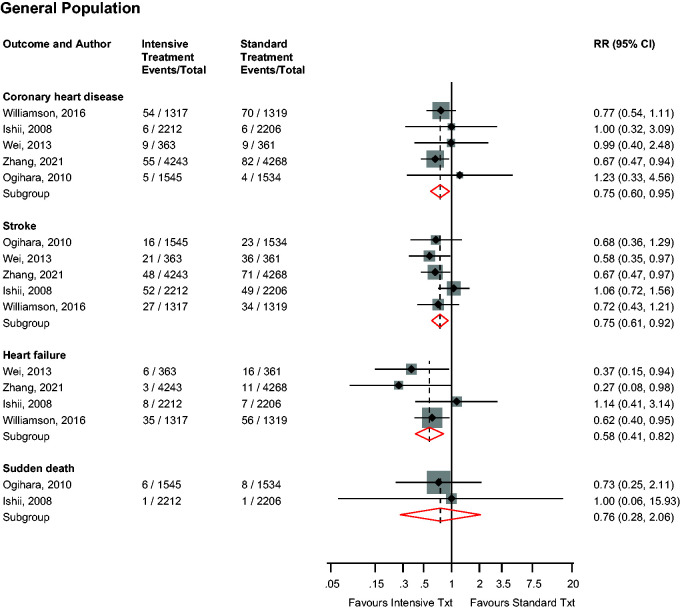
Risk for other cardiovascular outcomes in older people in the general population comparing intensive with standard blood pressure control.CI: confidence interval (bars); RR: risk ratio.

In the general population, intensive versus standard BP control was significantly associated with a reduced risk of CHD (*n* = 5 studies), stroke (*n* = 5 studies) and heart failure (*n* = 4 studies), with no significant association with sudden death (*n* = 2 studies): RRs (95% CIs) of 0.75 (0.60–0.95; *I*^2^ = 0%; 95% CI 0–79; *p* for heterogeneity = .81), 0.75 (0.61–0.92; *I*^2^ = 12%; 95% CI 0–82; *p* for heterogeneity = .34), 0.58 (0.41–0.82; *I*^2^ = 28%; 95% CI 0–73; *p* for heterogeneity = .25) and 0.76 (0.28–2.06), respectively (Figure 4). On exclusion of the trial by Zhang et al.,^19^ the RRs (95% CIs) for CHD, stroke, heart failure and sudden death were 0.83 (0.61–1.13), 0.79 (0.62–1.01), 0.62 (0.43–0.89) and 0.76 (0.28–2.06), respectively. Single study results showed no significant evidence of associations of intensive versus standard BP control with nonfatal MI, nonfatal stroke, coronary revascularisation and atrial fibrillation, but a decreased risk of nonfatal heart failure (Supplementary Appendix 5). No study evaluated these associations in patients with DM.

### Safety outcomes

**Figure 5.**
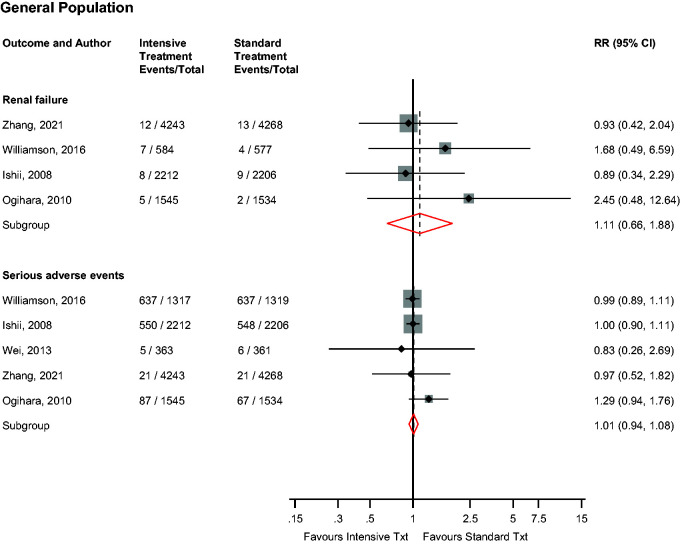
Risk for renal failure and serious adverse events in older people in the general population comparing intensive with standard blood pressure control.CI: confidence interval (bars); RR: risk ratio.

In the general population, intensive versus standard BP control was not associated with the risk of renal failure (*n* = 4 studies) or serious adverse events (such as gastrointestinal and respiratory symptoms, hypotension and abnormal laboratory findings) (*n* = 5 studies): RRs (95% CIs) of 1.11 (0.66–1.88; *I*^2^ = 0%; 95% CI 0, 85%; *p* for heterogeneity = .64) and 1.01 (0.94–1.08; *I*^2^ = 0%; 95% CI 0–79; *p* for heterogeneity = .62), respectively (Figure 5). On exclusion of the trial by Zhang et al.,^19^ the RRs (95% CIs) for renal failure and serious adverse events were 1.28 (0.64–2.57) and 1.01 (0.94–1.09), respectively. Single study results showed that intensive versus standard BP control was not associated with albuminuria (Supplementary Appendix 5). No study evaluated these associations in patients with DM.

### GRADE summary of findings

GRADE ratings for the relevant outcomes are reported in a summary of findings table in Supplementary Appendix 6. GRADE quality of the evidence ranged from moderate to low.

## Discussion

In this systematic review and meta-analysis of most contemporary RCT evidence, our results show that intensive BP control is associated with reduced risk of adverse cardiovascular outcomes in older hypertensive patients in the general population, with no significant evidence of associations with all-cause mortality risk or increased risk of renal failure or serious adverse events. The results were generally similar on exclusion of the trial which recruited patients aged 60–80 years.^19^ Limited trial data in older patients with DM showed that the rates of composite CVD/MACE did not differ between intensive and standard BP control groups. Meta-regression analysis revealed no significant difference between both populations in the risk of composite CVD/MACE conferred by intensive versus standard BP control, which was also consistent with results of one of the trials which compared results in patients with or without DM.^21^ The results of the interaction analysis need to be interpreted with caution given the limited number of studies available for this analysis.

This is the first systematic review and meta-analysis to attempt to assess and compare the benefits and harms of intensive versus standard BP control in older people with or without DM. We identified major evidence gaps, which is the lack of definitive trials of intensive BP control in older hypertensive patients with DM and should stimulate further work in this area. We included all published trial evidence based on the topic and evaluated a comprehensive list of efficacy and safety outcomes. We were also able to quantitatively summarise the available data, assessed the risk of bias in individual trials and rated the certainty of the outcome evidence using validated tools. There are several important limitations that deserve consideration. Given the limited evidence on patients with DM, head-to-head comparisons of the harms and benefits of intensive BP control could not be made between those with and without DM. For some of the outcomes, the number of events were too small, which are reflected in the imprecise CIs. There was some variability in study populations (inclusion and exclusion criteria), BP measurements, use of antihypertensives, thresholds for intensive and standard BP control and definition of outcomes. For instance, whereas some trials used an SBP target of <140 mmHg in the intensive BP control group, others used <120 mmHg or 110–129 mmHg. However, our estimates of heterogeneity showed zero to moderate heterogeneity across trials for the majority of outcomes. Finally, due to the limited data and lack of individual participant data, we could not explore for evidence of publication bias and treatment effects in relevant subgroups by age, sex and cardiovascular risk.

The findings of the current study cannot be directly compared with other studies, but some previous studies do deserve discussion. In a systematic meta-analysis of four RCTs that assessed the efficacy and safety of intensive BP lowering strategies in older (age ≥65 years) hypertensive patients, Bavishi et al. reported that intensive versus standard BP lowering was associated with reduction in MACE, CVD mortality and heart failure, with no significant differences in the incidence of MI, stroke, serious adverse events or heart failure.^18^ The current meta-analysis, which is based on additional RCTs, evaluated a comprehensive list of outcomes including all-cause mortality and the composite outcome of CVD plus renal dysfunction and showed that intensive BP control reduced the risk of CHD and stroke in older patients. A number of systematic reviews and meta-analyses have also been conducted in the topic area and have generally reported greater vascular protection with intensive BP control, but these were not based in older patients ≥65 years, and their control arms included standard BP control and placebo.^31,32^ In attempting to synthesise the sparse data on the harms and benefits of intensive BP control on older hypertensive patients with DM, we have demonstrated major evidence gaps and the urgent need for the conduct of larger definitive trials in this area.

The overall findings provide clear evidence on the benefits of intensive BP control in older hypertensive patients using an SBP threshold of <120 to <140 mmHg, which are consistent with recommendations from some guideline bodies.^2,13–15^ Whether this threshold applies to older patients with DM is not conclusive given the limited data. However, given that interaction analysis in one of the included trials^21^ as well as our meta-analysis revealed that the rate of major cardiovascular events due to intensive BP lowering was not significantly different between patients with and without DM, one could argue that intensive BP control may confer similar benefits for older patients with DM. However, these results are based on limited data and subgroup analyses, which are prone to false-positive results.^33^ Older patients with hypertension and DM are much more vulnerable to the effects of intensive BP control, given their multiple comorbidities in addition to frailty. It is therefore concerning that guideline-recommended BP targets for older adults with DM^2,34,35^ have been mostly based on inconsistent data extrapolated from the general population. This is also a major reason why some guidelines are not very clear on specific BP targets for older people with DM. The UK National Institute for Health and Care Excellence guideline for hypertension in adults recommends that clinic BP should be reduced to below 150/90 mmHg for adults with hypertension aged 80 years and over and states that clinical judgement should be used for people with frailty or multimorbidity.^13^ In the absence of clinical trial data on the benefits and harms of intensive BP control in older hypertensive patients with DM, management of hypertension in these patients should be individualised, tailored according to their health status and monitored closely.

## Conclusions

In older people with hypertension in the general population, intensive BP control (SBP <120 to <140 mmHg) decreased the risk of composite CVD/MACE, CVD mortality, CHD, stroke and heart failure, with no significant evidence of associations with all-cause mortality risk or increased risk of renal failure or serious adverse events. Limited trial data do not provide significant evidence that intensive BP control reduced the risk of composite CVD/MACE in older hypertensive patients with DM. Given the lack of RCTs of intensive versus standard BP control in older patients with DM, definitive trials are urgently warranted in this population group.

## Supplemental Material

sj-pdf-1-jrs-10.1177_01410768231156997 - Supplemental material for Intensive versus standard blood pressure control in older persons with or without diabetes: a systematic review and meta-analysis of randomised controlled trials Click here for additional data file. Supplemental material, sj-pdf-1-jrs-10.1177_01410768231156997 for Intensive versus standard blood pressure control in older persons with or without diabetes: a systematic review and meta-analysis of randomised controlled trials by Samuel Seidu, Harini Willis, Setor K Kunutsor and Kamlesh Khunti in Journal of the Royal Society of Medicine

## Declarations

### Guarantor

SS.

### Provenance

Not commissioned; peer reviewed by Julie Morris and Refai Showkathali.

### ORCID iD

Samuel Seidu https://orcid.org/0000-0002-8335-7018
