# Supplementary material for: Intensive versus standard blood pressure control in older persons with or without diabetes: a systematic review and meta-analysis of randomised controlled trials
Source: J R Soc Med. 2023 Feb 24;116(4):133–43. doi: 10.1177/01410768231156997 (PMC10164272; doi:10.1177/01410768231156997)
Supplement: sj-pdf-1-jrs-10.1177_01410768231156997 - Supplemental material for Intensive versus standard blood pressure control in older persons with or without diabetes: a systematic review and meta-analysis of randomised controlled trials [file sj-pdf-1-jrs-10.1177_01410768231156997.pdf]

SUPPLEMENTARY MATERIAL

**Appendix 1**	PRISMA checklist
**Appendix 2**	MEDLINE literature search strategy
**Appendix 3**	Outcomes reported by eligible trials
**Appendix 4**	Assessment of risk of bias using the Cochrane risk of bias tool
**Appendix 5**	Narrative synthesis results
**Appendix 6**	GRADE summary of findings

Appendix 1: PRISMA checklist

Section/topic	Item No	Checklist item	Reported on page No
**Title**			
Title	1	Identify the report as a systematic review, meta-analysis, or both	1
**Abstract**			
Structured summary	2	Provide a structured summary including, as applicable, background, objectives, data sources, study eligibility criteria, participants, interventions, study appraisal and synthesis methods, results, limitations, conclusions and implications of key findings, systematic review registration number	2
**Introduction**			
Rationale	3	Describe the rationale for the review in the context of what is already known	Introduction
Objectives	4	Provide an explicit statement of questions being addressed with reference to participants, interventions, comparisons, outcomes, and study design (PICOS)	Introduction
**Methods**			
Protocol and registration	5	Indicate if a review protocol exists, if and where it can be accessed (such as web address), and, if available, provide registration information including registration number	Methods
Eligibility criteria	6	Specify study characteristics (such as PICOS, length of follow-up) and report characteristics (such as years considered, language, publication status) used as criteria for eligibility, giving rationale	Methods
Information sources	7	Describe all information sources (such as databases with dates of coverage, contact with study authors to identify additional studies) in the search and date last searched	Methods
Search	8	Present full electronic search strategy for at least one database, including any limits used, such that it could be repeated	Appendix 2
Study selection	9	State the process for selecting studies (that is, screening, eligibility, included in systematic review, and, if applicable, included in the meta-analysis)	Methods
Data collection process	10	Describe method of data extraction from reports (such as piloted forms, independently, in duplicate) and any processes for obtaining and confirming data from investigators	Methods
Data items	11	List and define all variables for which data were sought (such as PICOS, funding sources) and any assumptions and simplifications made	Methods
Risk of bias in individual studies	12	Describe methods used for assessing risk of bias of individual studies (including specification of whether this was done at the study or outcome level), and how this information is to be used in any data synthesis	Methods
Summary measures	13	State the principal summary measures (such as risk ratio, difference in means).	Methods
Synthesis of results	14	Describe the methods of handling data and combining results of studies, if done, including measures of consistency (such as I^2 statistic) for each meta-analysis	Methods
Risk of bias across studies	15	Specify any assessment of risk of bias that may affect the cumulative evidence (such as publication bias, selective reporting within studies)	Methods
Additional analyses	16	Describe methods of additional analyses (such as sensitivity or subgroup analyses, meta-regression), if done, indicating which were pre-specified	Methods
**Results**			
Study selection	17	Give numbers of studies screened, assessed for eligibility, and included in the review, with reasons for exclusions at each stage, ideally with a flow diagram	Results, Figure 1
Study characteristics	18	For each study, present characteristics for which data were extracted (such as study size, PICOS, follow-up period) and provide the citations	Results, Table 1
Risk of bias within studies	19	Present data on risk of bias of each study and, if available, any outcome-level assessment (see item 12).	Results, Appendix 3
Results of individual studies	20	For all outcomes considered (benefits or harms), present for each study (a) simple summary data for each intervention group and (b) effect estimates and confidence intervals, ideally with a forest plot	
Synthesis of results	21	Present results of each meta-analysis done, including confidence intervals and measures of consistency	Results, Figures 2-5
Risk of bias across studies	22	Present results of any assessment of risk of bias across studies (see item 15)	Not applicable
Additional analysis	23	Give results of additional analyses, if done (such as sensitivity or subgroup analyses, meta-regression) (see item 16)	Not applicable
**Discussion**			
Summary of evidence	24	Summarise the main findings including the strength of evidence for each main outcome; consider their relevance to key groups (such as health care providers, users, and policy makers)	Discussion
Limitations	25	Discuss limitations at study and outcome level (such as risk of bias), and at review level (such as incomplete retrieval of identified research, reporting bias)	Discussion
Conclusions	26	Provide a general interpretation of the results in the context of other evidence, and implications for future research	Discussion
**Funding**			
Funding	27	Describe sources of funding for the systematic review and other support (such as supply of data) and role of funders for the systematic review	After Discussion

Appendix 2: MEDLINE literature search strategy

1	Age*.mp. (12764821)
2	Old*.mp. (1660684)
3	Elder*.mp. (301624)
4	exp Aged/ or Older.mp. (3644507)
5	Elderly.mp. (286632)
6	target blood pressure.mp. (1100)
7	goal blood pressure.mp. (307)
8	intensive blood pressure.mp. (592)
9	tight blood pressure.mp. (173)
10	strict blood pressure.mp. (265)
11	((randomized controlled trial or controlled clinical trial).pt. or randomized.ab. or randomised.ab. or randomly.ab. or trial.ab. or groups.ab.) not (exp animals/ not humans.sh.) (2893183)
12	1 or 2 or 3 or 4 or 5 (13166296)
13	6 or 7 or 8 or 9 or 10 (2380)
14	11 and 12 and 13 (970)
15	limit 14 to (humans and ("all aged (65 and over)" or "aged (80 and over)")) (561)
Each part was specifically translated for searching alternative databases.	

Appendix 3: Outcomes reported by eligible trials

Author, year of publication	Outcomes (no. of events)	Intensive BP control No. of events	Standard BP control No. of events
Ishii, 2008	Composite CVD/MACE<br>CVD mortality<br>Coronary heart disease<br>Stroke<br>Heart failure<br>Renal failure<br>Serious adverse events<br>Sudden death<br>Composite CVD plus renal dysfunction<br>All-cause mortality	26<br>6<br>6<br>52<br>8<br>8<br>550<br>1<br>86<br>54	28<br>4<br>6<br>49<br>7<br>9<br>548<br>1<br>86<br>42
Ogihara, 2010	Composite CVD/MACE<br>All-cause mortality<br>CVD mortality<br>Sudden death<br>Stroke<br>Myocardial infarction<br>Renal failure<br>Serious adverse events<br>Composite CVD plus renal dysfunction	32<br>24<br>11<br>6<br>16<br>5<br>5<br>87<br>30	37<br>30<br>11<br>8<br>23<br>4<br>2<br>67<br>38
Cushman, 2010	Composite CVD/MACE	20	23
Wei, 2013	Composite CVD/MACE<br>Stroke<br>Myocardial infarction<br>Heart failure death<br>CVD mortality<br>All-cause mortality<br>Serious adverse events	40<br>21<br>9<br>6<br>25<br>51<br>5	67<br>36<br>9<br>16<br>50<br>87<br>6
Williamson, 2016	Composite CVD/MACE<br>Myocardial infarction<br>Acute coronary syndrome<br>Coronary heart disease<br>Stroke<br>Heart failure<br>CVD mortality<br>Nonfatal myocardial infarction<br>Nonfatal stroke<br>Nonfatal heart failure<br>All-cause mortality<br>CKD outcome<br>Albuminuria<br>Serious adverse events	102<br>37<br>17<br>54<br>27<br>35<br>18<br>37<br>25<br>35<br>73<br>7<br>26<br>637	148<br>53<br>17<br>70<br>34<br>56<br>29<br>53<br>33<br>55<br>107<br>4<br>28<br>637
Zhang, 2021	Composite CVD/MACE<br>Stroke<br>Coronary heart disease<br>Heart failure<br>Coronary revasculirization<br>Atrial fibrillation<br>CVD mortality<br>All-cause mortality<br>Serious adverse events<br>Estimated GFR<30	147<br>48<br>55<br>3<br>22<br>24<br>18<br>67<br>21<br>12	196<br>71<br>82<br>11<br>32<br>25<br>25<br>64<br>21<br>13

Appendix 4: Assessment of risk of bias using the Cochrane risk of bias tool

	Randon sequence generation	*Allocation concealment*	*Blinding of participants & personnel*	*Blinding of outcome assessments*	*Incomplete outcome data*	*Selective reporting*	*Other bias*
**Ishii, 2008**	+	+	-	+	+	+	?
**Ogihara, 2010**	+	+	-	+	+	+	?
**Cushman, 2010**	+	+	-	+	+	+	?
**Wei, 2013**	+	?	-	+	+	+	?
**Williamson, 2016**	+	+	-	+	+	+	?
**Zhang, 2021**	+	+	-	+	+	+	?

+	Low risk of bias
?	Unclear risk of bias
-	High risk of bias

Appendix 5: Narrative synthesis results

Author, year of publication	Study	Population	Outcomes	Events/Total for intensive BP control group	Events/Total for standard BP control group	Results, risk estimates (95% CIs)
Williamson, 2016	SPRINT-Senior	Hypertension at increased risk for CVD	Nonfatal MI<br>Nonfatal stroke<br>Nonfatal HF<br>Albuminuria	37/1317<br>25/1317<br>35/1317<br>26/196	53/1319<br>33/1319<br>55/1319<br>28/177	0.69 (0.45-1.05)<br>0.68 (0.40-1.15)<br>0.63 (0.40-0.96)<br>0.96 (0.53-1.75)
Zhang, 2021	STEP	Systolic hypertension	Coronary revascularisation<br>Atrial fibrillation	22/4243<br>24/4243	32/4268<br>25/4268	0.69 (0.40-1.18)<br>0.96 (0.55-1.68)

BP, blood pressure; CI, confidence interval; CVD, cardiovascular disease; HF, heart failure; MI, myocardial infarction; SPRINT, Systolic Blood Pressure Intervention Trial; STEP, Strategy of Blood Pressure Intervention in the Elderly Hypertensive Patients

Appendix 6: GRADE summary of findings

**Intensive BP control compared to Standard BP control for Hypertension**					
**Outcomes**	**№ of participants (studies) Follow-up**	**Certainty of the evidence (GRADE)**	**Relative effect (95% CI)**	**Anticipated absolute effects**	
				**Risk with Standard BP control**	**Risk difference with Intensive BP control**
Composite CVD/MACE in general population	19,368 (5 RCTs)	⊕⊕⊕◯ Moderate[a]	**RR 0.71** (0.62 to 0.82)	49 per 1,000	**14 fewer per 1,000** (19 fewer to 9 fewer)
Composite CVD/MACE in DM	3,244 (2 RCTs)	⊕⊕⊕◯ Moderate[a]	**RR 0.85** (0.67 to 1.07)	44 per 1,000	**7 fewer per 1,000** (15 fewer to 3 more)
All-cause mortality in general population	19,368 (5 RCTs)	⊕⊕◯◯ Low[a,b]	**RR 0.84** (0.61 to 1.14)	34 per 1,000	**5 fewer per 1,000** (13 fewer to 5 more)
CVD mortality in general population	19,368 (5 RCTs)	⊕⊕⊕◯ Moderate[a]	**RR 0.65** (0.49 to 0.86)	12 per 1,000	**4 fewer per 1,000** (6 fewer to 2 fewer)
CHD in general population	19,368 (5 RCTs)	⊕⊕⊕◯ Moderate[a]	**RR 0.75** (0.60 to 0.95)	18 per 1,000	**4 fewer per 1,000** (7 fewer to 1 fewer)
Stroke	19,368 (5 RCTs)	⊕⊕⊕◯ Moderate[a]	**RR 0.75** (0.61 to 0.92)	22 per 1,000	**5 fewer per 1,000** (9 fewer to 2 fewer)
SAEs in general population	19,368 (5 RCTs)	⊕⊕⊕◯ Moderate[a]	**RR 1.01** (0.94 to 1.08)	132 per 1,000	**1 more per 1,000** (8 fewer to 11 more)

*__The risk in the intervention group__ (and its 95% confidence interval) is based on the assumed risk in the comparison group and the **relative effect** of the intervention (and its 95% CI).
BP, blood pressure; CHD, coronary heart disease; CI, confidence interval; CVD, cardiovascular disease; DM, diabetes mellitus; MACE, major adverse cardiovascular events; RR, risk ratio; SAE, serious adverse events

Intensive BP control compared to Standard BP control for Hypertension

Outcomes	№ of participants (studies) Follow-up	Certainty of the evidence (GRADE)	Relative effect (95% CI)	Anticipated absolute effects	
				Risk with Standard BP control	Risk difference with Intensive BP control

GRADE Working Group grades of evidence
High certainty: we are very confident that the true effect lies close to that of the estimate of the effect.
Moderate certainty: we are moderately confident in the effect estimate: the true effect is likely to be close to the estimate of the effect, but there is a possibility that it is substantially different.
Low certainty: our confidence in the effect estimate is limited: the true effect may be substantially different from the estimate of the effect.
Very low certainty: we have very little confidence in the effect estimate: the true effect is likely to be substantially different from the estimate of effect.

Explanations

a. High risk of bias in one domain
b. I-squared value of 72%
